# Feasibility of Using Low-Cost Dual-Frequency GNSS Receivers for Land Surveying

**DOI:** 10.3390/s21061956

**Published:** 2021-03-11

**Authors:** Natalia Wielgocka, Tomasz Hadas, Adrian Kaczmarek, Grzegorz Marut

**Affiliations:** 1 Institute of Geodesy and Geoinformatics, Wrocław University of Environmental and Life Sciences, 50-375 Wrocław, Poland; natalia.wielgocka@upwr.edu.pl (N.W.); adrian.kaczmarek@upwr.edu.pl (A.K.); grzegorz.marut@upwr.edu.pl (G.M.); 2 Institute of Navigation, University of Stuttgart, 70174 Stuttgart, Germany

**Keywords:** GNSS, low-cost, land-surveying, RTK, PPP, positioning accuracy

## Abstract

Global Navigation Satellite Systems (GNSS) have revolutionized land surveying, by determining position coordinates with centimeter-level accuracy in real-time or up to sub-millimeter accuracy in post-processing solutions. Although low-cost single-frequency receivers do not meet the accuracy requirements of many surveying applications, multi-frequency hardware is expected to overcome the major issues. Therefore, this paper is aimed at investigating the performance of a u-blox ZED-F9P receiver, connected to a u-blox ANN-MB-00-00 antenna, during multiple field experiments. Satisfactory signal acquisition was noticed but it resulted as >7 dB Hz weaker than with a geodetic-grade receiver, especially for low-elevation mask signals. In the static mode, the ambiguity fixing rate reaches 80%, and a horizontal accuracy of few centimeters was achieved during an hour-long session. Similar accuracy was achieved with the Precise Point Positioning (PPP) if a session is extended to at least 2.5 h. Real-Time Kinematic (RTK) and Network RTK measurements achieved a horizontal accuracy better than 5 cm and a sub-decimeter vertical accuracy. If a base station constituted by a low-cost receiver is used, the horizontal accuracy degrades by a factor of two and such a setup may lead to an inaccurate height determination under dynamic surveying conditions, e.g., rotating antenna of the mobile receiver.

## 1. Introduction

Global Navigation Satellite Systems (GNSS) are widely used in a variety of geoscience applications [1], including tectonic plate motion monitoring [2], surface deformations [3], landslide monitoring [4], seismology [5], as well as ionosphere [6] and troposphere [7] remote sensing. A GNSS receiver remains the core sensor in navigation systems of unmanned aerial vehicles [8] and autonomous vehicles [9]. Yet the GNSS have revolutionized not only geosciences but also land surveying [10], as they allow for determining position coordinates with centimeter-level precision in real-time or up to sub-millimeter accuracy in post-processing solutions.

Achieving precise and accurate position coordinates requires a combination of the three following aspects: availability of a well-defined reference frame; processing of carrier-phase measurements, use of an appropriate measurement model. Two general approaches can be distinguished, namely absolute and relative positioning. In absolute positioning, the reference frame for measurements is provided by satellite coordinates, while in relative positioning, a reference station has well-known coordinates. Therefore, the positioning accuracy of a GNSS directly depends on the accuracy of the coordinates of either the satellites or the reference station. The International GNSS Service (IGS, https://www.igs.org/) provides satellite orbits, clocks, and established control point networks with centimeter-level accuracy, in order to maintain national reference frames all over the world.

The relative positioning is commonly used in land surveying applications, in real-time post-processing. Real-Time Kinematic (RTK) or Network RTK (NRTK) modes allow for achieving centimeter-level accuracy in real-time [11] if there is a base station or a network of reference stations near the mobile receiver. A millimeter level accuracy can be achieved with a differential approach for long static surveys by means of networks of GNSS receivers separated by tens to hundreds of kilometers [12]. The dominant technique in absolute positioning is the Precise Point Positioning (PPP) [13], which works worldwide, even if high-precision results are still limited to static surveys and dual-frequency receivers [14]. Therefore, the choice of the measurement model should depend on the required positioning accuracy and availability of a local GNSS frame.

Even if both the well-defined reference frame and the chosen measurement model are available, the acquisition of carrier phase measurements remains critical. For many years, such a feature was limited to dual-frequency geodetic-grade receivers, whose high cost was the limiting factor for many applications [15,16]. Since low-cost single-frequency receivers capable of logging carrier phase measurements have become available, several studies aimed at investigating their positioning accuracy [17,18] and demonstrations of low-cost monitoring applications were performed [19,20]. Two main advantages of using low-cost receivers are the possibility of deployment in hazardous areas and logging a higher amount of position data [21]. Millimeter [15] to centimeter-level [22] accuracy can be achieved under favorable conditions [23], as the antenna is the key factor limiting the positioning accuracy [18]. Moreover, the ionosphere delay remains a major error source affecting single-frequency PPP [17] and long-baseline RTK [15]. Most of the low-cost single-frequency receivers available on the market do not meet their nominal performance in urban areas characterized by the multipath effect [24].

After the development of low-cost multi-frequency receivers, the ionosphere delay is no longer an issue and can be removed by combining the measurements obtained at two frequencies. Currently, two dual-frequency receivers are available on the market, namely u-blox ZED-F9P (https://www.u-blox.com) and SkyTraQ PX1122R (https://www.skytraq.com.tw). The former is capable of simultaneously tracking four GNSS (GPS, GLONASS, Galileo, and BeiDou). However, the positioning accuracy of low-cost receivers is worse than geodetic-grade receivers, thus increasing the convergence time of a real-time PPP solution [25]. If a low-cost patch antenna is used, it is needed to correct the carrier phase patterns [26]. Moreover, these receivers can be used for geodetic surveys in open sky conditions and short baselines [27].

Very few studies on the performance of a low-cost receiver combined with a low-cost antenna, used for precise relative and absolute positioning in static and kinematic mode, are available [28]. Although the manufacturers declare that their receivers enable a reliable positioning in RTK mode with a short convergence time, such a performance is limited to a favorable environment [24]. Therefore, this paper aims at evaluating the signal acquisition performance and accuracy of a low-cost dual-frequency multi-GNSS receiver, used for a relative (RTK and NRTK) and absolute static positioning in typical land-surveying applications, e.g., establishment of control points and cadastral surveying.

## 2. Materials and Methods

### 2.1. Low-Cost Receiver and Antenna

The u-blox C099-F9P evaluation board was used for the ZED-F9P high precision GNSS module. This board allowed flexible connectivity options (including USB and Arduino UNO R3 header) and easy receiver configuration through the free u-center software, provided together with the receiver. The receiver tracked GPS L1 C/A and L2 C, GLONASS L1 OF and L2 OF, Galileo E1-B/C and E5b, BeiDou B1I and B2I signals. The manufacturer declared a positioning accuracy of 1 cm + 1 ppm in RTK mode, with a baseline limited to 20 km.

The receiver was connected to a standard u-blox ANN-MB-00-00 patch antenna, having a circular ground plane (as recommended by the manufacturer). It was a Right Hand Circular Polarized (RHCP) dual-band antenna (L1 and L2/E5b/B2I). The manufacturer provided information on the typical maximum phase center offset (PCO) and phase center variations (PCV) only for GPS L1 and L2 frequencies. PCO was lower than 5 mm in the horizontal plane, lower than 8.9 mm, and 7.6 mm in the vertical plane for L1 and L2, respectively. PCV was lower than 5 mm and lower than 10 mm over all azimuth and elevations, for L1 and L2, respectively. The declared PCO and PCV values did not fit with the antenna calibration model developed by Krietemeyer et al. [26].

### 2.2. Reference Data and Measurements

Multi-GNSS 1 Hz measurements from IGS station WROC and from permanent stations of the national ASG-EUPOS system (http://www.asgeupos.pl) were used. Trimble R10 geodetic-grade receiver and antenna were used for performing measurements in the static mode (2 h-long sessions) on all control points and, then, the position data were processed through the automatic post-processing service POZGEO of ASG-EUPOS. Reference coordinates were provided in the Polish PL-2000 coordinate system, which was a Gauss-Krüger projection of coordinates determined in the European Terrestrial Reference System (ETRS) 89.

### 2.3. Experiment Setup and Processing Strategies

#### 2.3.1. Static Measurements

**Figure 1:**
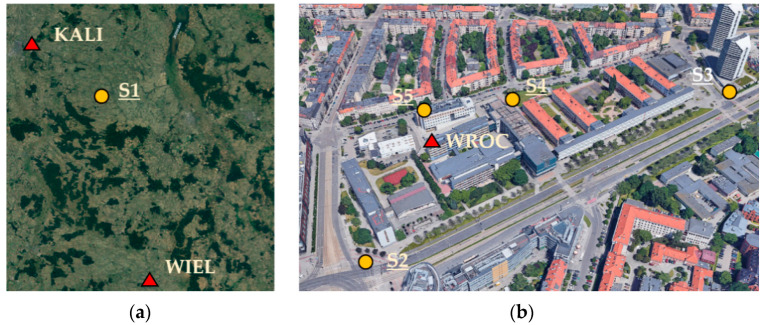
Location of control points and reference stations in (**a**) countryside and (**b**) urban areas.

Measurements were performed in five control points in two areas, i.e., one point (S1) in a rural area (on 21 September 2020) and four points (S2, S3, S4, and S5) in an urban area (on 25 October 2020). The rural area was in the countryside Suliszewice, near Sieradz city, Poland (Figure 1a), characterized by open-sky conditions. The point was measured for 5.5 h. The two nearest ASG-EUPOS stations were KALI (26 km far) and WIEL (50 km far). The urban area was in Wroclaw city, Poland, around the University campus, near buildings of different heights (up to 85 m), thus representing more challenging surveying conditions (Figure 1b). WROC station was located in the central part of this testing area. The longest baseline between a test point and the reference station was 385 m. Each point was measured for 1 h. Moreover, on 22 September 2020, a 24-h long measurement was performed near WROC station (roof of a building, open-sky conditions), which was used for analyzing the signal acquisition by a low-cost receiver.

In order to investigate the performance of a low-cost patch antenna and the signal acquisition by a low-cost receiver, the dependence of the carrier-to-noise ratio (C/N_0_) on the satellite elevation, GNSS and frequency was investigated. Then, the open-source RTKLib software (http://www.rtklib.com) was used for post-processing the data logged in the relative positioning. CSRS-PPP online service (https://webapp.geod.nrcan.gc.ca/geod/tools-outils/ppp.php) was used for the absolute positioning. Table 1 shows the details of both processing strategies. Moreover, in order to evaluate the impact of session length on the estimation of coordinates (both in relative and absolute positioning), the measurements in S1 were split into shorter and independent sessions, i.e., two sessions of 2.5 h, five sessions of 1 h, 11 sessions of 30 min and 22 sessions of 15 min. For relative positioning, the ambiguity fixing ratio was investigated. Finally, the uncertainty (i.e., the a posteriori standard deviation of estimated coordinates) and accuracy (i.e., the difference with respect to known coordinates) were evaluated for relative and absolute positioning, by using the measurements obtained during sessions of 1 h. In S1, the first one hour of observations was used, in order to have a fair comparison with measurements in the urban area. In the relative positioning, S1 coordinates were determined by using both the reference stations, in order to evaluate the impact of baseline length on the accuracy. The coordinates of S2, S3, S4, and S5 were determined with respect to the WROC station, without network adjustment, because a single point was measured in a time.

#### 2.3.2. RTK and NRTK

The performance of RTK and NRTK modes was evaluated in multiple configurations and the coordinates directly estimated by the mobile receiver were used. Firstly, a dual receiver configuration, in which both the base station and the mobile receiver were u-blox evaluation boards, was used. The antenna of the mobile receiver was mounted on a 0.93 m long arm, which spins around the antenna of the base station. The horizontal distance and height difference between the two receivers was analyzed in each epoch during 17 rotations and compared with a nominal value. As the orientation of the antenna of the mobile receiver with respect to the antenna of the base station was changing over time, patterns indicating either multipath effect or irregularities in the antenna phase center variation were searched.

Seven control points having known coordinates were selected and measurements in the following positioning modes were performed: (a) RTK using a geodetic grade receiver (WROC station) as a base station, (b) NRTK using network differential correction data transmitted by the Leica HxGn Smartnet service (https://www.smartnetleica.pl/smartnet/), based on Virtual Reference Station (VRS) concept, (c) RTK using a low-cost receiver as a base station. In each positioning mode, two measurement series were performed at each point. After every single measurement, the re-initialization of the receiver was carried out, in order for the ambiguities to be independently computed. The accuracy achieved by the low-cost receiver was analyzed in each positioning mode.

## 3. Results and Discussion

### 3.1. Carrier-to-Noise Ratio Analysis

**Figure 2:**
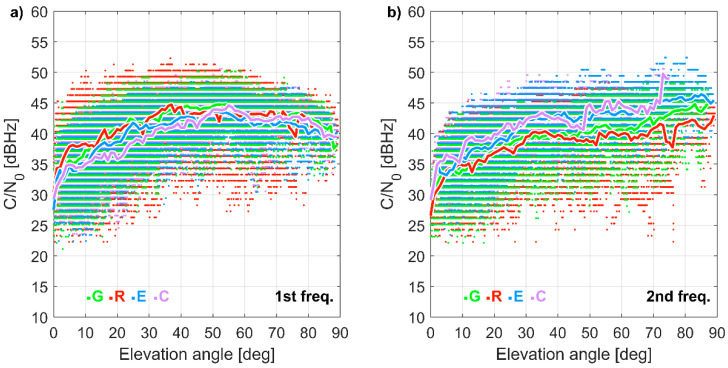
Carrier-to-noise ratio (C/N_0_) for (**a**) 1st and (**b**) 2nd frequency as a function of satellite elevation angle for the u-blox receiver. The dots indicate individual measurements, while the lines represent the elevation mean. 22 September 2020 (24 h). G, R, E, C stand for GPS, GLONASS, Galileo, and BeiDou, respectively.

A strong dependence of the C/N_0_ on satellite elevation was noticed (Figure 2). For the L1 frequency, the highest C/N_0_ ratios (26 to 53 dB Hz) were recorded for elevation angles between 35 and 70°. For higher elevation angles, C/N_0_ were higher than 26 dB Hz but remained lower than 50 dB Hz. Below 20°, a significant amount of measurements with C/N_0_ lower than 30 dB Hz was noticed. For the second frequency (L2/E5b/B2I), a nearly linear increase of the average C/N_0_ was recorded with the increasing elevation angle. Multiple C/N_0_ ratios lower than 30 dB Hz were noticed for elevation angles lower than 20°. By assuming that typical C/N_0_ ratios vary from 35 to 55 dB Hz with a minimum of around 28 dB Hz [29], a u-blox receiver combined with a low-cost antenna performed well, except for the low-elevation satellites.

**Figure 3:**
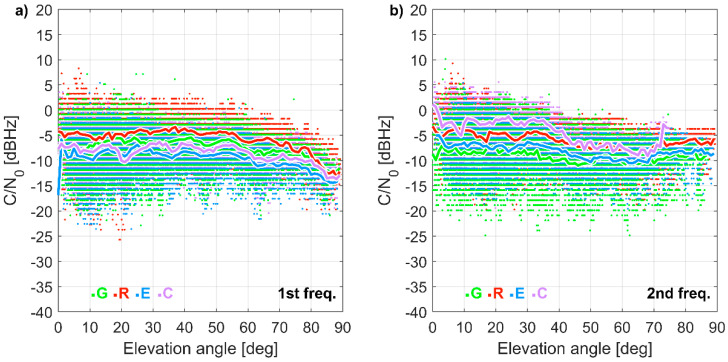
Carrier-to-noise ratio (C/N_0_) difference (u-blox minus a geodetic grade receiver at WROC station) for (**a**) 1st and (**b**) 2nd frequency as a function of satellite elevation angle: the dots indicate individual measurements, while lines represent elevation mean. 22 September 2020 (24 h).

Compared to a geodetic grade antenna and receiver (Figure 3), C/N_0_ ratios for a u-blox receiver resulted on average lower by 7.2 and 7.0 dB Hz for the first and second frequency, respectively. For the L1 frequency, 98.9% of measurements showed a lower C/N_0_ ratio by using the low-cost receiver, rather than the geodetic-grade receiver. For the second frequency, this percentage dropped to 93.7%. Although for most cases the C/N_0_ ratios were lower for the u-blox receiver, exceptional cases of stronger acquisition as well as the acquisition of signals weaker than 28 dB Hz, reflecting the implementation of a modern detection approach, which enhanced the acquisition of weak signals.

**Figure 4:**
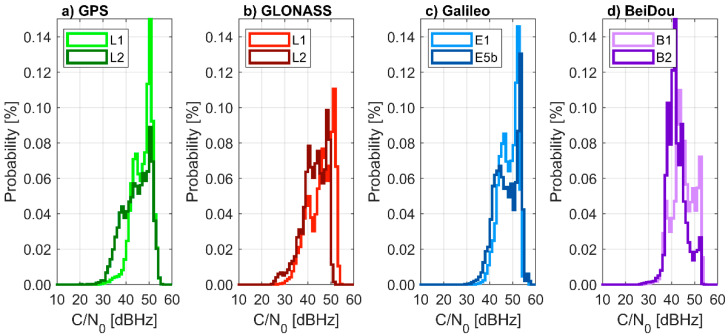
Histograms of carrier-to-noise ratio (C/N_0_) for each Global Navigation Satellite Systems (**a**) GPS, (**b**) GLONASS, (**c**) Galileo and (**d**) BeiDou and frequency, computed by using a u-blox receiver. 22 September 2020 (24 h).

The C/N_0_ differences between the two frequencies for the same satellite (Figure 4) corresponded to the difference in signal transmission energy, i.e., for GPS the L1 signal resulted from 3 to 7.5 dB higher than the L2 signal, for GLONASS the L1 signal was 6 dB higher than L2, for Galileo the E1 signal was 2 dB lower than E5b, for BeiDou the B1 signal was 4 dB higher than B2.

### 3.2. Static Positioning

#### 3.2.1. Session Length

The ambiguity fixing rate, i.e., the percentage of fixed carrier phase ambiguities in a session, was considered as a first quality indicator of the baseline solution. In the open sky environment (point S1), the average ambiguity fixing rate for the shortest baseline reached almost 80%, even though it varied from 43.3% to 97.9% for sessions of 15 min (Table 2). Therefore, it indicated that ambiguity fixing rate depends on satellite geometry and environmental conditions. The longer sessions were, the smaller the range of fixed ambiguities among sessions was. For the longest baseline, the average ambiguity fixing rate did not exceed 60%, and no ambiguity fixed was recorded during sessions of 15 min. Unresolved ambiguity resolution was mainly caused by atmospheric biases, which start playing a role for baselines exceeding 20 km [30].

**Figure 5:**
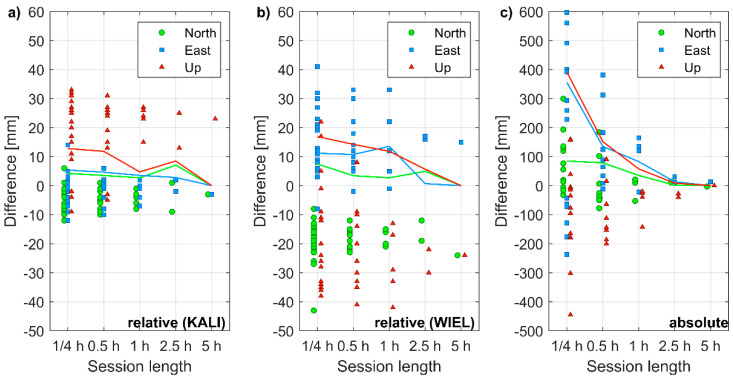
Coordinate differences of point S1 obtained for various session lengths: (**a**) in the relative positioning using the reference station KALI; (**b**) in the relative positioning using reference station WIEL; (**c**) in the absolute positioning.

For each session, the last fixed position was used for further accuracy analysis. The extended session length went in line with the increased accuracy of estimated coordinates (Figure 5). For the shortest baseline, the horizontal accuracy was better than 10 mm even during 1-h-long sessions. Yet, also during 15-min-long sessions, horizontal accuracy greater than 20 mm was achieved. Although the precision, i.e., the repeatability of the vertical component (Up), was similar for the horizontal coordinates (East and North), an average bias of +19 mm was recorded with respect to reference coordinates. This offset exceeded the sub-centimeter antenna PCO/PCV in the vertical plane, declared by the manufacturer. The scatter of coordinates estimated with the longest baselines was higher by a factor of 2 to 3. Even during the 5-h-long session, the horizontal and vertical accuracy exceeded 20 mm. Moreover, an average bias of −18 mm, +14 mm, and −20 mm was recorded for the North, East, and Up components, respectively.

For absolute positioning, the improvement of accuracy over time was even more evident than in the relative positioning. However, this was expected even for geodetic-grade receivers, because a significant change in satellite geometry was required to effectively de-correlate carrier phase ambiguities in PPP. After processing the 5 h-long sessions, the accuracy of −3 mm, +13 mm, and 0 mm was achieved for the North, East, and Up components, respectively.

#### 3.2.2. Positioning Confidence and Accuracy

**Figure 6:**
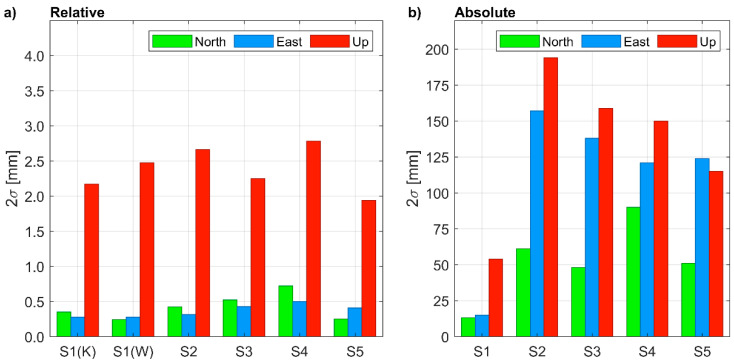
Coordinate uncertainty at 95% (2σ) confidence level in: (**a**) relative positioning (the reference station is KALI for S1(K), WIEL for S1(W), WROC for S2 to S5); (**b**) absolute positioning.

Estimated uncertainty of coordinates is another indicator of the quality of a positioning solution. For 1-h-long sessions and relative positioning, the uncertainty at a 95% confidence level was similar for all points and, surprisingly, did not depend on baseline length (Figure 6a). For S1 points measured in the open-sky conditions, as well as for points measured in the urban area (S1, S2, S3, S4, S5) similar 2σ uncertainties of less than 1 mm were achieved for horizontal coordinates and between 2 to 3 mm for the vertical component. With PPP (Figure 6b), the uncertainties resulted much higher and depended on the satellite visibility, i.e., they were significantly lower for S1 than for the other points. The decimeter level 2σ uncertainties achieved for the urban environment during 1-h-long sessions did not meet the requirements of precise positioning. Even in the open sky conditions, poor uncertainties (2σ) were achieved, i.e., they were lower than 20 mm and higher than 50 mm for the horizontal and vertical components, respectively.

**Figure 7:**
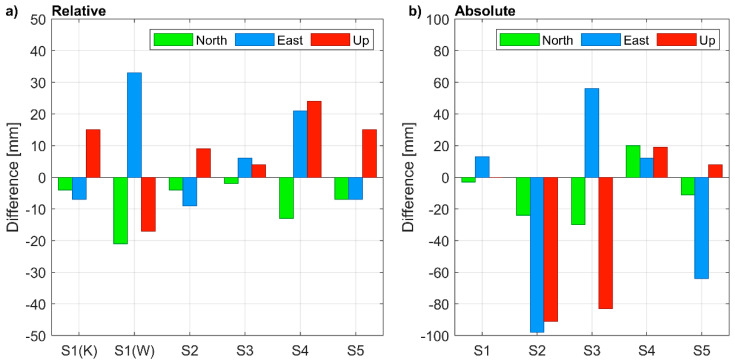
Coordinate differences from 1-h long sessions obtained in: (**a**) relative positioning (the reference station is: KALI for S1(K), WIEL for S1(W), WROC for S2 to S5); (**b**) absolute positioning.

The differences of coordinates with respect to reference data (Figure 7) resulted significantly higher in the relative positioning and much lower in the absolute positioning rather than corresponding uncertainties. Nevertheless, in the relative positioning, the centimeter-level accuracy was achieved both for the horizontal and vertical components. The corresponding root means square errors (RMSE) are 11, 17, and 15 mm for the North, East, and Up component, respectively. The worst accuracy was achieved for the S1 coordinates determined by using the 50-km-long baseline (WIEL). With PPP, coordinate differences resulted lower than 1 decimeter. Only for S1 was centimeter-level accuracy achieved, due to the open sky measurement conditions. The RMSE were 20, 58, and 56 mm, for the North, East, and Up components, respectively.

### 3.3. RTK and NRTK

#### 3.3.1. Baseline Precision

**Figure 8:**
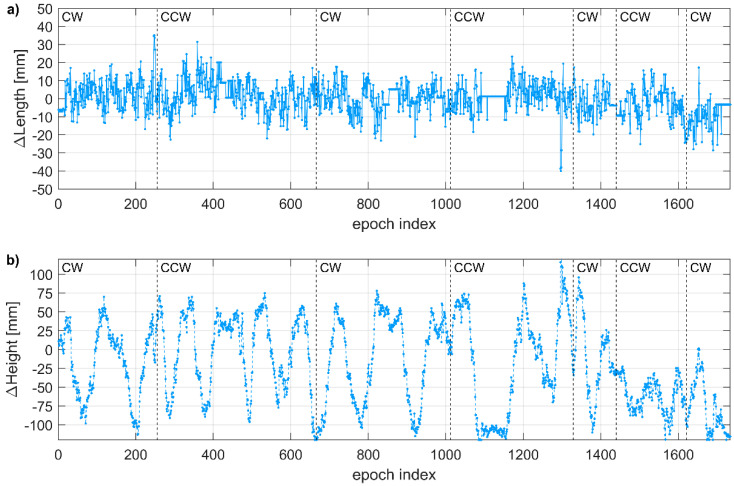
Positioning error in consecutive epochs: (**a**) for baseline length; (**b**) for the height difference. Dashed black lines indicate changes in the rotation direction (CW—clockwise, CCW—counter-clockwise).

From the experiment with a spinning mobile receiver, the baseline length, i.e., the horizontal distance between the base station and the mobile receiver, was continuously determined with high precision, i.e., the RMSE of length differences with respect to nominal baseline length was 8.6 mm (Figure 8a). Among 1734 registered epochs (2 Hz registration frequency), 7 epochs (0.7%) with length error exceeding 30 mm and 44 epochs (2.5%) with length error exceeding 20 mm were recorded. The highest positioning error was 52 mm.

The RMSE for the vertical component was 56.7 mm. For 46% of epochs, the positioning error for the height determination exceeded 5 cm, including 11% of epochs with errors exceeding 10 cm. Moreover, oscillations of the vertical component determination were noticed (Figure 8b). They were recorded during both rotation directions, i.e., clockwise (CW) and counterclockwise (CCW), and were not triggered by the change of rotation direction. All solutions were reported by the receiver as fixed, thus excluding the ambiguity fixing issues as a potential error source.

**Figure 9:**
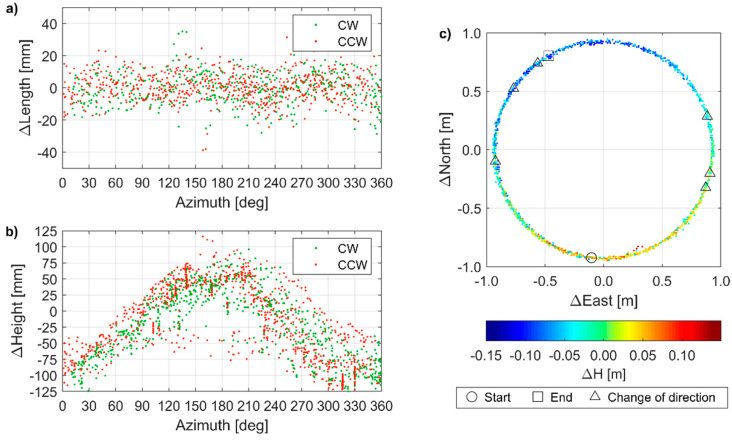
Positioning error: (**a**) as a function of azimuth for baseline length; (**b**) as a function of azimuth for height difference; (**c**) as a function of antenna locations for height difference (color-coded), with indicated changes in the rotation direction. CW—clockwise, CCW—counter-clockwise.

Solutions were assigned to rotation direction, i.e., CW and CCW. Thus, they were grouped into two disjunctive sets and their concentration was analyzed within a certain range of baseline azimuths (Figure 9). In both sets, small height determination errors were predominant for azimuths from 80 to 240°. Significantly underestimated heights (i.e., with height determination error exceeding 5 cm) were predominant for azimuths from 270 to 50°, while the significantly overestimated heights were recorded only within a limited azimuth range, i.e., between 155 and 231°. Therefore, inaccurate height determination was due to the orientation change of the antenna of the mobile receiver with respect to the antenna of the base station. This implied two possible error sources, i.e., missing PCO/PCV models or improper handling of the wind-up effect. The latter would result in the increase or decrease (depending on the rotation direction) of a measured carrier phase, thus producing accumulated height determination errors, as long as the rotation direction does not change. Since the height determination error did not accumulate (Figure 8b) but oscillated, i.e., they depended on the azimuth (Figure 9b), missing PCO/PCV models was identified as the error source. The opposite orientation of low-cost antennas (which corresponded to the azimuth around 0°) resulted in accumulated PCO/PCV error, which resulted in inaccurate height determination.

#### 3.3.2. Positioning Accuracy

**Figure 10:**
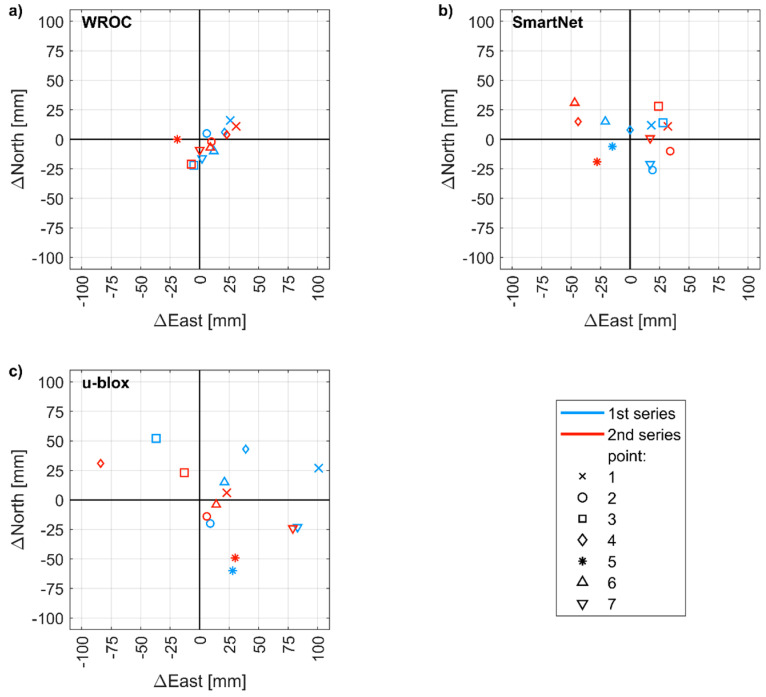
Positioning error (with respect to reference coordinates) of control points measured in two series by using: (**a**) WROC station as reference station; (**b**) Network Real-Time Kinematic (NRTK) SmartNet; (**c**) u-blox receiver as a base station.

In the last field experiment, the horizontal and vertical accuracy of RTK/NRTK was evaluated by using three different configurations in the urban environment. Each set of coordinates was determined from a fixed solution (as indicated by the receiver). The highest horizontal accuracy was achieved in RTK mode by using a geodetic-grade base station (Figure 10a). The RMSE of horizontal positioning error was 20 mm and the highest error was 33 mm. In the NRTK mode, the corresponding values were 32 mm and 56 mm (Figure 10b), which was in agreement with the horizontal positioning accuracy of 3 cm declared by the service provider.

In the configuration including a base station and a mobile receiver, both constituted by low-cost receivers (Figure 10b), the RMSE was 60 mm and the highest error reached 104 mm. The horizontal positioning precision, measured as the standard deviation of position errors, was 32 mm. The baselines were shorter than 0.5 km, thus achieving a positioning precision worse by a factor of 2 rather than the that declared by the manufacturer, i.e., 1 cm + 1 ppm.

**Figure 11:**
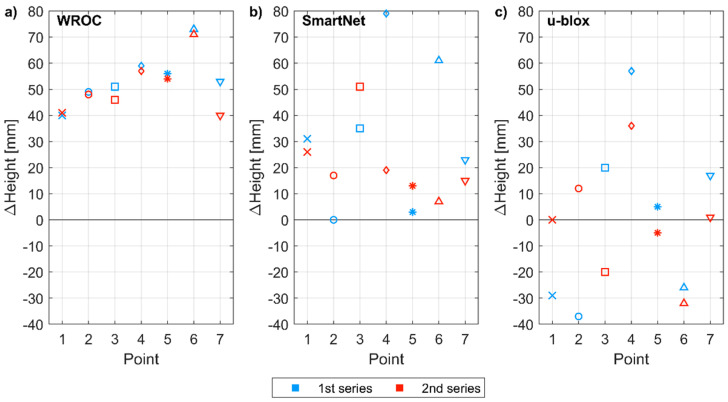
Positioning error for height (with respect to reference coordinates) of control points measured in two series by using: (**a**) WROC station as reference station; (**b**) NRTK SmartNet; (**c**) u-blox receiver as a base station.

The positioning error in height determination varied from −40 to +80 mm (Figure 11). For RTK with respect to the geodetic-grade base station, an average height error of 52 mm was recorded, but the standard deviation of height error was only 10 mm, indicating the high precision of the vertical component determination. In the NRTK mode, height was overestimated by 27 mm ± 23 mm. When two low-cost receivers are used for RTK, the positioning error for height was 0 mm ± 18 mm. The results indicate that both low-cost receivers have similar vertical PCO, which, however, is non-zero and causes overestimation of heights in NRTK or RTK modes with respect to a geodetic-grade base station.

## 4. Conclusions

The performance of the low-cost dual-frequency u-blox ZED-F9P receiver, connected to the low-cost ANN-MB-00-00 patch antenna, was evaluated. The current price of the hardware is lower than 250 EUR. C/N_0_ ratios in such a configuration resulted on average 7 dB Hz lower than those obtained by using geodetic-grade hardware. Still, the low-cost receiver performed well for elevation angles higher than 10°. Low C/N_0_ ratios for low elevation satellites were attributed to the simplified antenna construction, rather than the receiver itself. The C/N_0_ differences between GNSS and frequencies were due to varying signal transmission energy. Therefore, multi-GNSS signals acquisition with a low-cost receiver is feasible and has accepted characteristics, but it is worse than that achievable by using a geodetic-grade receiver.

In a static relative positioning under open sky conditions, almost 80% of fixed ambiguities were achieved with a 26 km long baseline. For the 50 km long baseline, the average ambiguity fixing rate did not exceed 60%, as the atmospheric error sources prevented ambiguity fixing. The fixing scores were c.a. 10% to 30% lower than those achievable by using a geodetic-grade receiver with baselines of similar lengths [31]. Nevertheless, a centimeter-level accuracy could be achieved in the relative positioning. The positioning accuracy increased with the increasing session length but it decreased with the increasing baseline length. For sessions longer than 1 h, a significant improvement was not observed, neither in the ambiguity fixing rate nor in the horizontal and vertical positioning accuracy. Longer sessions are justified for the absolute positioning using the PPP technique, in which the horizontal and vertical accuracy of few centimeters was achieved after 2.5 h of measurements.

In a more challenging surveying environment, i.e., an urban area, an hour-long static relative positioning allowed for achieving a horizontal (2D) and vertical accuracy of 20 and 15 mm, respectively. However, the estimated uncertainties at a 95% confidence level were an order of magnitude smaller. Contrary to baseline solutions, in the absolute positioning, the differences between estimated and known coordinates of control points were much lower than the estimated uncertainties. Differences between the estimated and true coordinates were within the ±10 cm range. Horizontal and vertical RMSE resulted in 62 and 56 mm, respectively. A distinct disagreement between accuracy and uncertainty was justified by stochastic models, which varied in the software and are not optimized for measurements performed by a low-cost receiver. Therefore, a limited confidence in the estimated positioning error, i.e., a posteriori standard deviation, is suggested.

In the RTK mode, a positioning precision exceeding that provided by the manufacturer, i.e., 1 cm + 1 ppm, was achieved. For a baseline shorter than 0.5 km and geodetic-grade base station, the horizontal error reached 33 mm (RMSE was 20 mm) and the vertical component (Up) was overestimated by 52 mm on average, with the highest error of 73 mm (RMSE was 53 mm). The measurements performed by using two low-cost receivers, of which one as a mobile receiver and the other as a base station, led to further degradation of the positioning precision. Still, the positioning accuracy was high, i.e., the RMSE was 60 and 26 mm for the horizontal and vertical components, respectively. In the NRTK mode, the positioning accuracy resulted between the two aforementioned RTK configurations and was in agreement with the horizontal accuracy of 3 cm declared by the NRTK service provider. Last but not least, inaccurate height determination was noticed in RTK surveying by using two low-cost receivers, of which the mobile receiver spins around the base station. A source of this issue was identified in accumulating PCO/PCV errors of the used low-cost antennas, as the highest errors mostly occurred when antennas were in the opposite orientation.

Similar to low-cost single-frequency receivers [25], low-cost dual-frequency receivers do not meet their nominal performance in urban areas. However, the positioning accuracy achieved in static and RTK/NRTK modes justifies their use in land surveying applications, such as cadastral surveying and mapping, which require sub-decimeter horizontal accuracy. Static positioning using low-cost receivers (both base station and mobile receiver), which achieves baseline accuracy better than 1 cm, is suitable for monitoring applications, including hazard and atmosphere monitoring. Although low-cost solutions, which are tailored for land-surveying, are not available on the market yet, this market is expected to rapidly grow soon.

## Figures and Tables

**Table 1:** Processing strategies for the relative and absolute positioning.

	Relative Positioning	Absolute Positioning
Software/service	RTKLib	CSRS-PPP
GNSS	GPS + GLONASS + Galileo	GPS + GLONASS
Satellite orbits and clocks	MGEX Final (COM)	MGEX Final (COM)
Technique	double-differencing	PPP
Frequencies	L1 + L2/L5	Ionosphere-free from L1 + L2
Measurement frequency	1 Hz	1 Hz
Elevation mask	10°	7.5°
Satellite PCO/PCV	igs14.atx	igs14.atx
Antenna PCO/PCV	none	none
Troposphere delay	Saastamoinen	estimated
Ambiguities	fixed	float

**Table 2:** Statistics of fixed ambiguities [%] in the relative mode for point S1.

Session Length	Reference Station: KALI			Reference Station: WIEL		
	min	max	Average	min	max	Average
15 min	43.3	97.9	78.2	0.0	94.1	41.3
30 min	61.2	95.3	79.5	10.5	85.3	53.0
1 h	71.5	88.8	79.5	35.4	86.7	55.6
2.5 h	78.4	81.2	79.8	46.2	68.2	57.2
5 h	79.9	79.9	79.9	58.0	58.0	58.0
